# Annual Address

**Published:** 1878-08

**Authors:** A. Wilkes Smith

**Affiliations:** President Kentucky State Dental Association


### ANNUAL ADDRESS.

BY A. WILKES SMITH, PRESIDENT KENTUCKY STATE DENTAL ASSOCIATION.

GENTLEMEN OF THE KENTUCKY STATE DENTAL ASSOCIATION.—Another year has passed and gone since we last met and exchanged friendly greetings. It must thrill each and every one of us with pleasure when we refer back to the pleasant yearly meetings of this society. We might speak of it as the annual reunion of a large family—fathers and sons all in the profession of our choice.

Webster defines a profession as a calling, a vocation, a known employment.

We are here to-day as the representatives of the youngest, yet the strongest and the grandest of all the acknowledged professions. I state that it is no egotism for me to claim this for the profession or specialty of dentistry, and while we stand with uncovered heads and bow with reverence to the old institutions of learning that have fostered the sciences and arts, and to the great men who have devoted their lives to scientific research: Our hearts must swell with pride when we know that these old timed honored institutions have accorded to us our true position as the grand success of the nineteenth century in the founding of this American institution.

It was reserved for America, the land of freedom, the youngest and the grandest of all nations, to become the mother of this profession of ours.

In relating all the peculiar characteristics of an American institution—progress, ingenuity, scientific research, and to use our American phrases, push, go a-headitiveness, vim, etc., I need not recount the history of this profession—the gray haired fathers still live and can relate with more vivid recital the rise and progress of our calling, their success and their failures, the illustrous past and the glorious future. I will just here take occasion to refer to the grand old man that meets with us from year to year in our State Association, encourages us by his presence and teaching us words of wisdom. I refer to Dr. William H. Goddard, of Louisville.

The avocation of dentistry is peculiarly an American institution. What Europe was to art, literature, law and medicine, so is America to-day to the profession of dentistry. By way of recapitulation, I will here give a brief summary of the progress of the collateral sciences in our country and abroad. In Chemistry, Boyle's, Mariotte's, Ampere's and Avogadro's researches in the physical properties of gases and vapors have received their verification in the liquefaction of the hitherto permanent gases, oxygen, hydrogen, nitrogen air and others. The new chemistry of to-day stands firmer through these discoveries, and if no other results follow the experiments of M. M. Pieteta and Cailletets, chemistry claims their names as pioneers, and science generally is indebted to them. Molecular matter is receiving attention by both physicists and chemists and the molecules of many elementary substances may be considered as magnitudes where dimensions are approximately known. In astronomy, on the other hand, much is being done. Spectrum analysis is daily sorting out the constituents of our heavenly bodies. New asteroids, or more properly speaking, planetoids, have been discovered and two satelites of interest to astronomers found by our own countryman, Hall, of Washington. In physics America is again well to the front, and all Europe bends its ear to our telephone. Electricity from its birth to the present epoch in its history is eminently American, and Franklin, Morse, Edison and Bell form a long line of ancestry to which we point with pride. The phonograph, no less wonderful than the telephone, is a physical reality of 1878.

In medicine and surgery we find that the past few years have developed many useful and valuable discoveries, among which may be found, first, the plaster of paris jacket for the cure of spinal curvatures, brought before the profession by Prof. Sayer, of New York. It possesses great advantage over the awkward and clumsy braces of the past; the cheapness of which places it within reach of the poor, among whom the disease is more frequently found.

Second, the transfusion of blood, while it does not effect a cure when the disease is organic, (only prolonging life), may be used very advantageously and with happy results in certain cases of exhaustion from loss of blood, etc., the strength thus artificially supplied acting until the natural processes are efficient.

Third, the tendency of dermatologists to bestow less attention to the myriads of classifications and sub-classifications of eruptions, and turning more time to etiology, which throws more light upon the treatment.

The perfecting of the sphygmograph which by giving the trace and shape of the pulse as is peculiar to and characteristic of certain maladies, furnishes a means of establishing the diagnosis beyond all doubt which hitherto remained in obscurity.

It might be proper for me to take a more extended view of the past few years in the history of science, American skill and industry; the field being so broad that I am compelled to confine myself to a few notes only.

American society is composed of a people of various callings, tastes, etc., all busy bees in the great hive of industry, the grand aristocracy of merit, industry and intelligence. We find in this society men of all the various avocations all striving to be elevated to the highest pinnacle of human grandeur by their meritorious acts and deeds, Some honor their calling whilst others hope that their calling may honor them. The latter may be designated as the drones in the hive. Foremost amongst the former we shall expect to find the dentist. It will take but a retrospective glance to show the rapid strides of the profession of dentistry. Scarcely half a century has passed since its foundation as a specialty, a grand profession. Whilst it has held to some false doctrines and to much that might be called empirical, we now find it, in less than half a century, composed of men well versed in the collateral sciences, men of thought, culture and intelligence.

It will be well for us as members of the Kentucky State Dental Association to take a retrospective glance at the history of our own State. As stated before the gray haired fathers still live and can recount the events of the past, therefore I shall not attempt to make mention of any period except the last few years that are fraught with so much healthy and abundant fruit. I will refer back to the formation of this Association.

In February 6th, 1868, an act was passed by the Legislature of the State of Kentucky granting the charter of this society, a little over ten years ago. In last March an act was passed by the Legislature of the same state making it a criminal offence to practice dentistry in this State without a proper qualification. These qualifications are sufficient to insure the public that the person complying with them can practice his calling with a degree of scientific skill and ability. Whilst the law does not cover the broad space that all intelligent dentists know the case demands, in the clearance of the field of the putrid carcasses that still cling to the profession, still we hope that it may have a purifying influence in the future. And greater to be thanked is the public recognition of the line of demarkation between the quack and the true professional gentleman. We may truly say that the last ten years of our labors as a society have been rewarded by this act of a just and intelligent legislature. As evidences of progress in this regard, I will note that this law was twice before presented before the legislature of our state and voted down. To-day it is one of the recognized laws of our land.

The past ten years of our existence can be referred to with pride in other matters of progress that have steadily and healthfully become established. We all well know that the conservatism that exists in our minds to-day is to be attributed to that steady progress in scientific research that characterises this association. To-day we find all differences in modes of practice bridged by broad conservatism, so that the mingling of the thoughts and ideas of our compeers are cherished as golden drops from the great fountain of knowledge, and we all know that we are benefited by this intermingling of thoughts and exchange of greetings,

The past year has developed a strong healthful progress in the science of dentistry, and while we may not endorse every thing new as adding to this progressive age, it will be well for us to give all a fair consideration, endorsing all that is worthy and discarding all that is worthless, and if as "skillful surgeons we are compelled to cut beyond the wound to make the cure complete," no true man devoted to his profession, to its glory and its honor will wince at the operation.

Then let the Kentucky State Dental Association resolve itself into an investigating committee, accepting that which is good, and refusing that which is false. Many new and useful instruments and appliances have been introduced, prominent are the diamond disks, drills, etc., the perfecting of the electric plugger, improvements in chairs and other apparatus. Some new theories have been introduced in practice, prominent is the "new departure or plastic fillings as the best method of saving teeth." The high professional standing of the gentlemen supporting this theory insures it a place in the deliberations of this body. To dental pathology and therapeutics much attention has been devoted by Dr. J. Foster Flagg, of Philadelphia. His researches in the Dental Cosmos are worthy of attention,

In the application of forces in the regulation of teeth some attention has been paid of late by the introduction of jack screws, swivel jacks, etc., known as the positive system. I would recommend to the members of our profession a more thorough acquisition of knowledge in physical laws. The late improvements in the use of the microscope as a means of diagnosis in various affections, both in medicine and dentistry, claim our admiration and attention. The student in the science of dentistry can not afford to dispense with this acquisition to his outfit. In this connection I will mention the practice of filling roots of teeth with lead, introduced by Dr. Peabody several years ago, but verified at a late date in its therapeutic effects, in its forming the salt of lead sulphide which acts antiseptically on decayed and decaying nitrogenous matter. The practice is highly recommended in obstinate cases of alveolar abscess, etc.

The standard of dental education is steadily being elevated to that position of which we may well feel proud. Since the leading universities of our land have created departments in this science.

The abuses of dental institutions of learning might occupy some space here, but as my mind recalls no institution the course of which, if well pursued, will not benefit the student, I will only urge a higher standard for all, and bid them God speed in their attempts to educate young men in a worthy calling.

The domestic or home education of the dentist might occupy our attention and with much profit to all. All of us will admit that the student armed with his diploma from a dental college has only begun his lesson when he is aware that he knows little or nothing at all. It is the hard school of experience. research and patient study that fit us for our calling. While there is much to be learned in other relations not professional, the successful dentist of the future should be a thorough gentleman. Gentlemen are born not made so by circumstances, therefore I will only mention some of the acquisitions necessary in order to maintain the dentist as the gentleman of society.

The legitimate, laudable ambition of all men who persue a calling is to obtain, wealth, position and fame. We should also place in the foremost rank, that it is the duty of all men to do all the good they can for society and the cause of humanity. To secure the former some disregard the latter. The American dentist stands in a position where he can acquire all. He can acquire wealth in abundance sufficient to satisfy the most ambitious. The avenues are open—ours is a lucrative profession. That wealth is necessary to the establishment of professional standing, must be evident to all. The old saying that "nothing is as successful as success" may in a degree be applied here. But we say, all of any profession do not acquire wealth. Admit they do not; all men of ability in a profession like ours, living in an intelligent community, may acquire a competency and sufficient to sustain him in the circle to which he justly belongs.

The art of money making should be studied as a text book by the dentist. He should be thoroughly posted as to the monetary affairs of the country, and should thoroughly understand finance, commerce and trade. Above all he should well know the old adage, "Time is money," and never allow a moment to be wasted or trifled away. It is not the object of this address to plan a prescribed road to wealth for the dentist to pursue, but only to urge the necessity of that acquisition in order to secure the grand success to which all should aspire.

The dentist as the gentleman of society is also a teacher in society, and since his social relations are of the highest order, it will be an easy task for him to educate his social circle to a proper appreciation of professional skill and ability. But how are we to reach the masses that we do not come in contact with in any capacity except through a casual professional call?

Dental associations have worked hard to elevate the members of our calling up to the proper standard of professional integrity. They have utilized all laudable means to establish a thorough code of ethics, jurisprudence and etiquette which is applauded by the world. But does the world understand the meaning of all this? Have we educated our communities to have a proper respect for that which we hold so sacred?

This is a subject to be well digested before acting. The question right here to be considered is the best method of educating our patients to the proper standard of professional appreciation. Whilst we discountenance all advertising in the way of circulars, hand bills, etc., as below the dignity of the true professional gentleman, it would be well for this association to issue a small hand book on dental hygiene, to be placed in the hands of our patients. This treatise to be thorougly arranged and criticised by a select committee, and adopted by this association. The matter should be a plain matter of fact paper, with directions to patients as to the treatment of their children's teeth and the care of their own teeth whilst not in the care of the dentist. The demand for this must be evident to all when we find how our operations suffer for want of care between the stated times that these patients call, and also the ignorance of parents in regard to children's teeth. The yearly sacrifice of the six year old molars is a startling proof of the need of advice in this direction.

Trusting, gentlemen, that the same broad and liberal views that have characterised the former meetings of this association, and that science, the profession and ourselves may be benefited by these meetings, I close these remarks.

